# The effect of a diabetes prevention program on dietary quality in women with previous gestational diabetes

**DOI:** 10.1186/s12905-019-0788-0

**Published:** 2019-07-03

**Authors:** Sharleen O’Reilly, Vincent Versace, Mohammadreza Mohebbi, Siew Lim, Edward Janus, James Dunbar

**Affiliations:** 1 UCD Institute of Food and Health, University College Dublin, Dublin, Ireland; 2 Deakin Rural Health, School of Medicine, Deakin University, Geelong, Australia; 3 Biostatistics unit, Faculty of Health, Deakin University, Geelong, Australia; 4 Monash Centre for Health Research and Implementation, Monash University, Clayton, Australia; 5 General Internal Medicine Unit, Western Health and Department of Medicine, Melbourne Medical School – Western Precinct, University of Melbourne, Melbourne, Australia

**Keywords:** Gestational diabetes, Diabetes prevention program, Diet quality, Program engagement

## Abstract

**Background:**

Women with gestational diabetes have low diet quality. We evaluated the effectiveness of a group-based lifestyle modification program for improvement of dietary quality in women with previous gestational diabetes predominantly within their first postnatal year.

**Methods:**

Women were randomised to intervention (*n* = 284) or usual care (*n* = 289). Dietary data was collected at baseline and twelve months using a food frequency questionnaire and recoded into the Australian Recommended Food Score (ARFS). Mixed model analyses investigated the intervention effect on ARFS (per-protocol-set (PPS) excluded women without the minimum intervention exposure).

**Results:**

Baseline mean total ARFS was low (31.8 ± 8.9, maximum score = 74) and no significant changes were seen in total ARFS (Cohen’s D = − 0.06). 2% reduction in alcohol for intervention (0.05, 0.26) compared with − 1% for usual care (Odds ratio: 0.68; 95%CI 0.46, 0.99). Dairy ARFS sub-category significantly improved (low fat/saturated fat foods) in the intervention group over time compared with usual care for the PPS analysis (dairy + 0.28 in intervention (95%CI 0.08, 0.48) compared with + 0.02 in usual care (95%CI -0.14, 0.18) (group-by-treatment interaction *p* = 0.05, Cohen’s D = 0.14)).

**Conclusions:**

Engaging with the intervention improved aspects of diet quality that aligned with minimum intervention exposure, but the total diet quality remains low. Further research is needed to improve diabetes prevention program engagement.

**Trial registration:**

Australian New Zealand Clinical Trials Registry ANZCTRN12610000338066, April 2010.

## Background

Gestational diabetes mellitus (GDM) is defined as any degree of glucose intolerance with onset or first recognition during pregnancy. According to the International Association of the Diabetes and Pregnancy Study Groups (IADPSG) criteria, about one in five pregnancies are complicated with GDM [1] and this will lead to increased risk of caesarean section, macrosomia and pre-eclampsia [2]. In the long term, GDM also increases the woman’s risk of developing type 2 diabetes (T2DM). The conversion rate of GDM to T2DM varies across geographical regions and increases with postpartum follow-up years; relative risks range from 1.3 to 47.3 [3] but the rates for Australia are estimated to fall between approximately 26 to 6% [4–6]. Diabetes prevention programs are effective in reducing the incidence in diabetes in the general population [7, 8]. A recent systematic review found that even small weight loss could lead to reduction in diabetes incidence in high-risk populations, with every kilogram weight loss associated with 43% lower diabetes odds [9]. In postpartum women with previous GDM, lifestyle interventions tend to produce smaller effects with a mean weight loss of about 1 kg [10, 11]. However, small weight changes in this group (< 2 kg) do modify the woman’s risk of T2DM and cardiovascular disease development [12, 13]. Several diabetes prevention programs in women with previous GDM have shown small but significant reduction in T2DM incidence and insulin resistance [11]. Participants in such programs are generally younger and healthier than typical older at-risk populations but a small, earlier intervention effect can potentially yield greater benefits in terms of health over the longer term - if improvements are sustained.

Most diabetes prevention interventions in women after GDM include dietary modification [11]. Achieving a greater number of quantitative dietary goals (e.g. less than 30% energy from fat, less than 10% energy from saturated fat, and increased fibre) are associated with an incremental reduction in diabetes risk [14]. To achieve these defined macronutrient goals, specific individualised meal plans are required yet these are rarely done in routine disease prevention practice because long term adherence is challenging [15]. Highly structured lifestyle advice is a particularly poor fit for reproductive age women, resulting in high attrition rates despite greater weight loss efficacy [16]. The main reason for this attrition being the competing priorities between self-care, work and needs of the family at this life-stage alongside the challenges of tiredness and a lack of time [17]. Thus, achieving qualitative dietary changes are potentially more likely for diabetes prevention in postpartum women over achieving set macronutrient intakes and describing the resulting changes in terms of food groups and food types may be a better reflection of dietary advice uptake.

Diet quality has been associated with T2DM risk in the general population across various ethnicities, which are not explained by body weight changes [18, 19]. Women with previous GDM have lower diet quality compared with those without [20, 21]. Even small diet quality improvements has been shown to improve glycemic management during GDM [22], although the effect in the postpartum period is not known. In a 20-year follow-up of women with previous GDM increased diet quality was associated with less weight gain suggesting potential T2DM prevention benefit [23, 24]. Despite the importance of diet quality in T2DM prevention, little is known about the effect on diet quality of diabetes prevention programs in postpartum women. This secondary analysis aims to explore the effect of a postpartum diabetes prevention program intervention (Mothers After Gestational Diabetes in Australia, MAGDA) [25] on diet quality as measured using the Australian Recommended Food Score (ARFS) in women with previous GDM.

## Methods

### Study population

Women with recent GDM were randomized to a multicentre, prospective, open randomized controlled trial in the Australian states of Victoria and South Australia. The study protocol was previously published, as well as the primary outcomes of the study [25–27]. In brief, women 18 years and older with GDM diagnosed using the Australasian Diabetes in Pregnancy Society (ADIPS) criteria in their most recent pregnancy were eligible for inclusion. The diagnosis of GDM at any time in pregnancy was based on any one of the following values: fasting plasma glucose 5.1–6.9 mmol/L; 1-h post 75 g oral glucose load ≥10 mmol/L; 2-h 75 g oral glucose load 8.5–11.0 mmol/L. All women were within 12 months postpartum at commencement of the study. Any women with pre-existing diabetes, cancer, severe mental illness, substance abuse, myocardial infarction in the preceding three months, difficulty with English; involvement in another post-natal intervention trial; and pregnancy at post-natal baseline testing or at any point during the 12 months of study involvement were excluded.

### Recruitment

Recruitment occurred in antenatal clinics after the diagnosis of GDM was made at approximately 28 weeks. Eligible women were provided with a pre-paid envelope containing the patient information and consent form to return within 4 weeks. If they were not returned within that timeframe follow-up contact was made with the women. Additional women were recruited through the Australian National Gestational Diabetes Register (NGDR) using relevant postcodes in Victoria and South Australia. Hospital records were searched for women with recent GDM and referrals from a private consultant obstetrician were also conducted in South Australia to increase recruitment. After written informed consent was obtained, baseline testing occurred. Eligible women were randomised following baseline diabetes screening. Permuted block randomisation was used and it was stratified by recruitment location and method using the MADGA-DPP management database. A sequence number and assignment code were revealed to the randomisation office at Deakin University and eligible women were allocated to intervention or usual care. Deakin University’s Human Research Ethics Committee (2010–005) maintained the primary ethical review, partner organisations maintained other ethical review procedures and these are listed in table one of the MAGDA-DPP protocol paper [27]. The study was approved by the relevant ethics committees and registered as an RCT (Australian New Zealand Clinical Trials Registry ANZCTRN 12610000338066). The study adhered to CONSORT guidelines.

### Intervention

The intervention consisted of an individual session completed in the woman’s home and five group sessions conducted at community settings close to the woman’s home. The individual session included personalised risk identification, goal setting and diabetes prevention through lifestyle modification. The group sessions covered healthy eating, physical activity and overall wellbeing. The Finnish Diabetes Prevention Study goals were used for this intervention, which were ≤ 30% total energy from total fat intake, ≤10% total energy from saturated fat, ≥15 g dietary fibre per 1000 kcals, ≥30 min moderate to vigorous physical activity per day and ≥ 5% weight loss [28]. The first group session covered diabetes and diabetes risk factors, reducing saturated fat and family-focused activities to achieve this, and the setting and review of personalised goals. The second session covered reduction of total fat, postpartum weight management, reducing the fat content in whole family’s diet, and the setting and review of personalised goals. The third session covered increasing fibre, healthier food shopping, getting more fibre into the whole family’s diet, and the setting and review of personalised goals. The fourth session covered healthier meal planning, negotiating stressful situations around food choice with family members, mindful eating, good sleep hygiene, and the setting and review of personalised goals. The final session covered postnatal depression awareness, stress management, maintenance of dietary and physical activity behaviour change, review of personalised goals and longer-term goal setting. Sessions lasted approximately 120 min and were held at fortnightly intervals. Two follow-up phone calls occurred at 3 months and 6 months after the active intervention period to reinforce behaviour change. The control group received usual care within their local setting. All women were followed up at 12 months after the baseline measurements.

### Health and dietary assessment

Blood samples were collected by the study nurse/phlebotomist and analyzed by a private pathology company. Height, weight, waist circumference and blood pressure were measured using standard protocols. Fasting venous blood samples was analyzed for triglycerides, total cholesterol, low-density lipoprotein cholesterol and high-density lipoprotein cholesterol, HbA1c, and fasting glucose and 2-h glucose tolerance as previously described [25]. Baseline and 12 months survey data included: demographics (breastfeeding, parity, education, employment status, cultural background; baseline only); Food Frequency Questionnaire (FFQ) [29]; Active Australia Questionnaire [30]; diet and physical activity self-regulation and self-efficacy; Multi-dimensional Scale of Perceived Social Support; Assessment of Quality of Life 8D; Patient Health Questionnaire; and health status (smoking status and history of diabetes, myocardial infarction, cancer, and mental disorders; baseline only).

Dietary analysis data were obtained using the Cancer Council of Victoria FFQ at baseline and 12 months. The dietary intakes were recoded using the Australian Recommended Food Score (ARFS) [31], which provides a qualitative assessment of the dietary intake and higher scores indicate a greater adherence to the Dietary Guidelines for Australians [32]. The ARFS in nine categories were summed to provide a maximum total ARFS of 74 for the best diet quality. Within the vegetables category, a maximum score of twenty-two could be achieved. There were twenty-one named types of vegetables and they each received a score of one if one or more servings were consumed in a week, and zero if less than that. An additional score of one is awarded for frequency of vegetable intake four or more servings daily. Within the fruit category a maximum score was fourteen could be achieved. There were thirteen types of fruit and they each received a score of one if one or more servings were consumed in a week, and zero if less than that. An additional score of one is awarded for frequency of fruit intake two or more servings daily. The grains category had a maximum score of fourteen. Consumption of high fibre white bread, wholemeal bread, rye bread, multigrain bread, all-bran, sultana bran/fibre plus/branflakes, weet-bix/vitabrits/weeties, rice, pasta/noodles, vegemite/marmite/promite, porridge, muesli, cornflakes/nutrigrain/special K each scored a one if consumed at least once a week, zero if not consumed. An additional score of one is awarded for consuming four or more slices of bread per day. The protein category had a maximum score of fourteen, which was split into the following sub-categories: nuts and legumes (total score of seven); meats, eggs and poultry (total score of five); and fish (total score of two). The nuts and legumes sub-category scored one for the consumption of one or more servings of nuts, peanut butter, baked beans, soy beans/tofu, soya milk, chickpeas, lentils per week, and zero if less than that. The meat and poultry sub-category was scored one if one to four servings of each of the following were consumed in a week: beef, veal, lamb, pork, chicken, up to two eggs and zero for any amount above or below this. The fish subcategory was scored one if one to four servings of each of the following were consumed in a week: fish (steamed/grilled/baked) and canned fish (salmon/tuna/sardines) and zero for any amount above or below this. The dairy category had a maximum score of seven. Consuming more than 500 ml milk per day received a score of one, zero if not. The consumption of reduced fat or skim milk, yoghurt, ricotta/cottage cheese, low-fat cheese received a score of one if one or more servings each were consumed in a week, and zero if less than that. Consuming cheese and ice-cream less than once per week received a score of one, zero if more than that. The fats category awarded a score of one if polyunsaturated spread, monounsaturated spread, or no fat spread were used, zero if any other used. The alcohol category was scored one for the consumption of less than once per month and up to four days/week of beer/wine/spirits and zero if outside those limits. Consuming more than two standard drinks per day received a score of zero and one if inside those limits.

### Statistical analyses

Statistical analysis was performed using SPSS version 22. Baseline participant characteristics are shown as summary measures. All participants randomized to the study (‘intention to treat’; ITT, *n* = 573) and those meeting the ‘per protocol set’ (PPS, *n* = 331) were analysed. The definition of the PPS was decided a priori. 14% of usual care (40/289) and 12% of intervention (35/284) women became pregnant during the trial and were excluded from ITT and PPS analyses. Women who were lost to contact (*n* = 52) or withdrew (*n* = 19) were also excluded from ITT and PPS analyses. Protocol deviations (hence exclusion from PPS) included assessments outside of the specified time window (*n* = 2); control group allocation but received intervention (n = 1); other postnatal intervention participation (*n* = 3); exclusion criteria met during intervention (T2DM (*n* = 12)). Women who did not attend a minimum of one group and the individual session (*n* = 78, 49% intervention participants) were also excluded from the PPS as they did not meet the minimum intervention exposure.

Linear mixed model analyses was used for all ordinal scale endpoints implementing the residual maximum likelihood (REML) estimation method to cope with missing values. The models include fix effect of intervention allocation and measurement time as nominal factors and the two-way interaction between treatment allocation and measurement time. In this setting the two-way interaction estimates the between-group differential change from baseline at 12-month follow-up (i.e. intervention effect). Within-participant autocorrelation was taken into account by assuming an exchangeable covariance pattern for repeated measures. Significance of the F-test for the treatment allocation and measurement time interaction is reported as well as t-tests for the within-group changes over time and the between-group differences at each time. Effect size (Cohen’s d) was calculated based on observed data with effects being interpreted as small (.20–.49), medium (.50–.79), or large (≥.80) [33]. Alcohol and fat scores were dichotomised endpoints and generalised linear mixed models with binomial distribution and logit link were used to analyse them. The model structure was similar to the linear mixed models analysis and odds ratio (OR) and 95% CI for the treatment allocation and measurement time was reported as intervention effect. All statistical tests were conducted at the two-sided 5% significance level with no adjustments for multiplicity of either endpoints or comparisons. Significance was achieved where *P*-value was ≤0.05. The sample size required to achieve an effect size of ≥0.27 in FPG over 12 months based on the GGT-DPP study, which was a six session, group-based Australian diabetes prevention program intervention [34], (assuming mean difference between intervention and control groups of 0.14 mmol/L and within group standard deviation of 0.5 mmol/L), using a two-sided 5% significance level and 80% power, was 574 (287 in each arm). A 25% attrition rate was added to the sample size calculated. A post hoc power analysis using the available sample size and observed standard deviation (i.e. SD = 7) for AFRS total score showed that the study has more 80% power to detect effect size (defined as standardised mean difference) 0.25 or larger.

## Results

### Characteristics of the women

573 women were randomised into intervention (*n* = 289) and control (*n* = 284). At baseline (see Table 1), the participants were well matched but had higher BMIs, larger waist circumferences, less physical activity than the average for Australian women within this age group [35]. These differences were not reflected in their metabolic health as the mean HbA1c was 34 mmol/mol (5.34%) and only 10% had impaired glucose tolerance and 2% impaired fasting glucose.

**Table 1:** Baseline characteristics for MAGDA study participants by treatment condition

Outcomes	Control (*n* = 289)	Intervention (*n* = 284)	Total (*n* = 573)	Significance (*P*-value)
Height (cm)				
N	289	284	573	
Mean (SD)	161.7 (7.2)	161.6 (6.9)	161.7 (7.1)	0.91
Weight (kg)				
N	289	284	573	
Mean (SD)	74.6 (20.3)	76.7 (20.0)	75.6 (20.2)	0.22
BMI (kg/m2)				
N	289	284	573	
Mean (SD)	28.4 (6.7)	29.2 (6.9)	28.8 (6.8)	0.13
Waist Circumference (cm)				
N	289	283	572	
Mean (SD)	90.4 (14.5)	92.1 (14.4)	91.2 (14.5)	0.15
Age (years)				
N	287	281	568	
Mean (SD)	33.6 (5.1)	34.1 (5.3)	33.8 (5.2)	0.20
Systolic BP (mmHg)				
N	289	284	573	
Mean (SD)	111.6 (12.8)	113.2 (14.0)	112.4 (13.4)	0.17
Diastolic BP (mmHg)				
N	289	284	573	
Mean (SD)	70.8 (10.1)	72.2 (10.7)	71.5 (10.4)	0.10
Diabetes perceived risk				
N	287	281	568	
Mean (SD)	5.4 (2.6)	5.7 (2.3)	5.6 (2.5)	0.20
Australian Quality of Life score				
N	284	272	556	
Mean (SD)	0.8 (0.2)	0.8 (0.1)	0.8 (0.1)	0.13
Tertiary education	237 (83%)	237 (84%)	474 (84%)	
Low Income	57 (20%)	71 (26%)	128 (23%)	
Current Smoker	21 (7%)	11 (4%)	32 (6%)	
Fulltime employed	49 (17%)	46 (16%)	95 (17%)	
Breastfeeding Initiated	259 (90%)	238 (85%)	497 (88%)	
Parity				
1	126 (44%)	127 (45%)	253 (45%)	
2	97 (34%)	97 (35%)	194 (34%)	
3+	64 (22%)	57 (20%)	121 (21%)	
Cultural Background by geo-region				
Asia	110 (38%)	113 (40%)	223 (39%)	
Australia and New Zealand	58 (20%)	73 (26%)	131 (23%)	
Aboriginal and Torres Strait Islander	0 (0.0%)	1 (0.4%)	1 (0.2%)	
Fasting plasma glucose (mmol/L)				
N	289	284	573	
Mean (SD)	4.7 (0.5)	4.8 (0.5)	4.8 (0.5)	0.03*
OGTT – 2 h (mmol/L)				
N	289	282	571	
Mean (SD)	5.6 (1.6)	5.5 (1.7)	5.5 (1.6)	0.91
HbA1c (%)				
N	289	283	572	
Mean (SD)	5.3 (0.5)	5.3 (0.4)	5.3 (0.4)	0.95
Total Cholesterol (mmol/L)				
N	289	284	573	
Mean (SD)	5.1 (0.9)	6.1 (1.0)	5.1 (0.9)	0.34
Triglycerides (mmol/L)				
N	289	284	573	
Mean (SD)	1.2 (0.7)	1.2 (0.7)	1.2 (0.7)	0.15
HDL (mmol/L)				
N	289	284	573	
Mean (SD)	1.54 (0.35)	1.47 (0.42)	1.50 (0.39)	0.03*
LDL (mmol/L)				
N	288	284	572	
Mean (SD)	3.1 (0.9)	3.0 (0.9)	3.0 (0.9)	0.40

Data presented as N, mean (standard deviation) and N (percentage)

Independent T-tests and chi-squared analysis

*Abbreviations*: *BMI* Body Mass Index, *BP* Blood Pressure, *OGTT* Oral Glucose Tolerance Test, *HDL* High Density Lipoproteins, *LDL* Low Density Lipoproteins

*significant values *P* ≤ 0.05

Baseline dietary intakes data showed the average energy intake was 7868 kJ/day (1881 kcal/d). Detailed intake data are shown in Table 2. Total fat intakes were 38% and saturated fat intakes were 16% of total energy intake. Dietary fibre was 10.9 g/1000 kcal. Total AFRS scores were 31.8 out of 74 pointing to a relatively low dietary quality (see Table 2). There were no significant differences seen at baseline for any dietary intake data.

**Table 2:** Baseline dietary intake and ARFS scores for diet quality in MAGDA study split by intervention status

Outcome	Control^a^ (*n* = 287)	Intervention^a^ (*n* = 281)	Total^a^ (*n* = 568)	Significance^b^ (*P*-value)
Dietary Intakes				
Energy (kJ/day)	7947 (3600)	7787(3217)	7868 (3414)	0.58
All Fat (g/day)	80.7 (39.8)	79.9 (36.6)	80.3 (38.3)	0.81
Saturated Fat (g/day)	32.8 (17.1)	32.4 (15.1)	32.6 (16.1)	0.78
Polyunsaturated Fat (g/day)	11.3 (6.0)	11.3 (5.7)	11.3 (5.9)	0.99
Monounsaturated Fat (g/day)	29.1 (14.7)	28.8 (14.2)	28.9 (14.4)	0.83
Protein (g/day)	96.6 (49.2)	93.2 (44.9)	94.9 (47.2)	0.40
Carbohydrates (g/day)	198.2 (89.6)	193.9 (77.8)	196.1 (84.0)	0.54
Fibre (g/day)	20.5 (8.7)	20.4 (8.2)	20.5 (8.5)	0.88
Australian Recommended Food Score				
Fruit	5.3 (3.1)	5.8 (3.3)	5.6 (3.2)	0.12
Vegetables	13.6 (4.1)	13.4 (4.7)	13.5 (4.4)	0.53
Grains	4.2 (1.7)	4.3 (1.8)	4.2 (1.7)	0.48
Meat, Poultry and Eggs	2.6 (1.5)	2.6 (1.5)	2.6 (1.5)	0.77
Nuts and Legumes	1.7 (1.3)	1.8 (1.3)	1.8 (1.3)	0.45
Fish	0.9 (0.8)	0.8 (0.8)	0.9 (0.8)	0.30
Total Protein	5.2 (2.2)	5.2 (2.2)	5.2 (2.2)	0.90
Dairy	2.8 (1.1)	2.8 (1.1)	2.8 (1.1)	0.85
Fat	0.4 (0.5)	0.4 (0.5)	0.4 (0.5)	0.83
Alcohol	0.1 (0.3)	0.1 (0.3)	0.1 (0.3)	0.79
Total ARFS	31.7 (8.4)	32.0 (9.4)	31.8 (8.9)	0.64

^a^ Data are presented as mean (Standard deviation). ^b^ *P*=Values resulting from T-Tests carried out between control and intervention groups

### Metabolic outcomes in ITT and PPS analyses

The metabolic outcomes are described in detail in O’Reilly et al. [25] but in summary for the co-primary endpoints, the ITT analysis showed the intervention group’s mean weight loss was 0.23 kg (95% CI -0.89, 0.43) compared with weight gain of 0.72 kg (95% CI 0.09, 1.35) in the usual care group (change difference 0.95 kg, 95% CI -1.87, − 0.04; group by treatment ITT interaction *P* = 0.04) over 12 months. The intervention group’s mean waist circumference reduction was − 2.24 cm (95% CI -3.01, − 1.42) compared with − 1.74 cm (95% CI -2.52, − 0.96) in the usual care group (change difference − 0.50 cm, 95% CI -1.63, 0.63; group by treatment ITT interaction *P* = 0.389) over 12 months. The intervention group’s mean increase in fasting blood glucose was 0.18 mmol/L (95% CI 0.11, 0.24) compared with an increase of 0.22 mmol/L (95% CI 0.16, 0.29) in the usual care group (change difference − 0.05 mmol/L, 95% CI -0.14, 0.05; group by treatment ITT interaction *P* = 0.331) over 12 months. No other statistically significant results were identified across the primary and secondary endpoints when using the F-test of the group by time interaction – a result that was consistent for both ITT and PPS.

### Diet quality in ITT analyses

The ITT analysis did not find any significant difference with intervention over time in the dietary quality as measured using AFRS total score (see Table 3). The nuts and legumes sub-category was significantly decreased (*p* = 0.04) driven by change within the intervention group over time (*p* = 0.01, Cohen’s D = − 0.19). Other small changes, some positive (improved dairy sub-score in intervention group over time, *p* <  0.01, Cohen’s D = 0.14) and some negative (decreased grain sub-score in both groups over time, p <  0.01, Cohen’s D = − 0.07), were seen over time or by treatment group but not in the overall model analysis. There was a significant 2% reduction in alcohol for the intervention compared with − 1% in usual care group (p = 0.04).

**Table 3:** Two-way table of predicted means (SEM) for the Australian Recommended Food Score categories by treatment condition and time (Intention To Treat analysis, ITT). Differences (p-value) over time and between treatments are shown in the table margins

ARFS categories	Control^#^ (*n* = 228)	Intervention^#^ (*n* = 205)	Difference	Effect size^§^
Fruit (*p* = 0.66) ^a^				
Baseline	5.39 (0.19)	5.79 (0.19)	0.40 (0.14)	–
12 Months	5.75 (0.21)	6.03 (0.21)	0.28 (0.35)	0.12 (−0.7, 0.31)
Differential change	0.36 (0.057)	0.255 (0.22)	−0.12 (− 0.66, 0.42)^†^	− 0.06 (− 0.25, 0.13)
Vegetables (*p* = 0.44) ^a^				
Baseline	13.63 (0.26)	13.38 (0.26)	0.25 (0.50)	–
12 Months	13.56 (0.28)	13.58 (0.29)	0.02 (0.96)	0.03 (−0.16, 0.22)
Differential change	−0.07 (0.77)	0.20 (0.42)	0.27 (−0.41, 0.94)^†^	0.05 (−0.13, 0.25)
Grains (*p* = 0.72) ^a^				
Baseline	4.18 (0.10)	4.28 (0.10)	0.11 (0.46)	–
12 Months	3.78 (0.11)	3.82 (0.12)	0.04 (0.79)	0.06 (−0.13, 0.25)
Differential change	−0.4 (< 0.01) *	− 0.47 (< 0.01) *	− 0.06 (− 0.40, 0.27)^†^	−0.07 (− 0.27, 0.11)
Protein				
Meat, Poultry and Eggs (*p* = 0.84) ^a^				
Baseline	2.63 (0.09)	2.59 (0.09)	−0.04 (0.75)	–
12 Months	2.62 (0.09)	2.56 (0.10)	−0.07 (0.63)	0.01 (−0.18, 0.20)
Differential change	0.00 (0.97)	−0.03 (0.75)	−0.03 (− 0.28, 0.23)^†^	−0.04 (− 0.24, 0.14)
Nuts and Legumes (p = 0.04) ^a,^ *				
Baseline	1.73 (0.08)	1.82 (0.08)	0.09 (0.40)	–
12 Months	1.75 (0.08)	1.61 (0.09)	−0.14 (0.24)	− 0.13 (− 0.32, 0.06)
Differential change	0.03 (0.72)	−0.20 (0.01) *	− 0.23 (− 0.45, − 0.01)^†^	−0.19 (− 0.38, 0.00)
Fish (*p* = 0.511) ^a^				
Baseline	0.41 (0.03)	0.35 (0.03)	−0.07 (0.10)	–
12 Months	0.40 (0.03)	0.37 (0.03)	−0.03 (0.50)	−0.02 (− 0.21, 0.17)
Differential change	− 0.01 (0.77)	0.02 (0.53)	0.08 (− 0.6, 0.21)^†^	0.10 (− 0.09, 0.29)
Total protein (p = 0.38) ^a^				
Baseline	5.23 (0.13)	5.22 (0.13)	−0.02 (0.93)	–
12 Months	5.22 (0.44)	5.03 (0.15)	−0.19 (0.34)	−0.07 (− 0.27, 0.11)
Differential change	− 0.01 (0.94)	−0.19 (0.19)	− 0.18 (− 0.57, 0.22)^†^	−0.10 (− 0.29, 0.09)
Dairy (*p* = 0.06) ^a^				
Baseline	2.81 (0.07)	2.83 (0.07)	0.01 (0.87)	–
12 Months	2.81 (0.07)	3.02 (0.08)	0.22 (0.04) *	0.25 (−0.44, 0.05)
Differential change	− 0.01 (0.93)	0.19 (< 0.01) *	0.20 (− 0.00, 0.41)^†^	0.14 (− 0.06, 0.33)
Total Score (*p* = 0.94) ^a^				
Baseline	31.72 (0.52)	31.98 (0.52)	0.26 (0.73)	–
12 Months	31.89 (0.57)	32.10 (0.58)	0.21 (0.80)	0.08 (−0.11, 0.28)
Differential change	0.17 (0.72)	0.12 (0.80)	−0.05 (−1.40, 1.29)^†^	− 0.04 (− 0.23, 0.16)
Alcohol (*p* = 0.86) ^a^				
Baseline	33 (11.5)	30 (10.7)	−0.02 (CI:-0.15, 0.11)	–
12 Months	24 (10.9)	20 (9.9)*	−0.03 (CI:-0.18, 0.13)	0.68 (0.46, 0.99)
Differential change	−0.01 (0.14)	−0.02 (0.15)	0.94 (CI:0.48, 1.85)^$^	
Fats (*p* = 0.12) ^a^				
Baseline	102 (35.5)	104 (37.0)	0.02 (CI:-0.07, 0.10)	–
12 Months	83 (37.6)	96 (47.1)	0.10 (CI:0.00, 0,19)	1.11 (0.60, 2.09)
Differential change	0.02 (0.09)	0.10 (0.10)	1.41 (CI:0.92, 2.15)^$^	

^a^ *p*-value for the F-test of Time by Treatment interaction

*Significant *p*-value (*p* ≤ 0.05)

^#^ Mean (SD) for continuous scores, and number (percent) for dichotomised scores

§Cohen’s D effect size (95% CI) for continuous scores, and OR (95% CI) for dichotomised scores

^†^ Between-group (intervention versus placebo) differential change from baseline was estimated from the two-way interaction between intervention allocation and measurement time from (generalised) linear mixed model

^$^ Odds ratio for comparing between-group (intervention vs placebo) change from baseline estimated from generalised linear mixed model’s two-way interaction between intervention allocation and measurement time

### Diet quality in PPS analyses

The PPS analysis defined a priori the major protocol violations, which included insufficient intervention exposure (minimum of one individual and one group session). There was no significant change in total ARFS between intervention and usual care over time (Table 4). However, the dairy intake sub-category achieved significance for improvement in the intervention group over time compared with usual care (*p* = 0.05). The difference for the dairy sub-category score between groups at 12 months was 0.39 (*p* <  0.01).

**Table 4:** Two-way table of predicted means (SEM) for the Australian Recommended Food Score categories by treatment condition and time (Per Protocol Set analysis, PPS)

ARFS categories	Control (*n* = 211)	Intervention (*n* = 120)	Difference
Fruit (*p* = 0.75) ^a^			
Baseline	5.24 (0.22)	5.52 (0.29)	0.28 (0.44)
12 Months	5.60 (0.22)	5.78 (0.29)	0.18 (0.62)
Differential change	0.36 (0.06)	0.26 (0.30)	−0.10 (−0.72, 0.52)
Vegetables (*p* = 0.49) ^a^			
Baseline	13.58 (0.29)	13.53 (0.39)	−0.06 (0.91)
12 Months	13.53 (0.30)	13.75 (0.39)	0.22 (0.65)
Differential change	−0.05 (0.83)	0.22 (0.485)	0.28 (−0.51, 1.06)
Grains (*p* = 0.69) ^a^			
Baseline	4.20 (0.12)	4.56 (0.16)	0.36 (0.07)
12 Months	3.80 (0.12)	4.07 (0.16)	0.28 (0.16)
Differential change	−0.41 (< 0.01) *	−0.49 (< 0.01) *	− 0.08 (− 0.48, 0.32)
Protein			
Meat, Poultry and Eggs (*p* = 0.42) ^a^			
Baseline	2.62 (0.10)	2.93 (0.13)	0.32 (0.05)
12 Months	2.62 (0.10)	2.81 (0.13)	0.19 (0.25)
Differential change	0.00 (0.98)	−0.12 (0.32)	−0.13 (− 0.43, 0.18)
Nuts and Legumes (*p* = 0.09) ^a^			
Baseline	1.69 (0.09)	1.70 (0.12)	0.02 (0.92)
12 Months	1.74 (0.09)	1.53 (0.12)	−0.21 (0.16)
Differential change	0.05 (0.51)	−0.17 (0.11)	−0.22 (− 0.49, 0.04)
Fish (*p* = 0.851) ^a^			
Baseline	0.86 (0.06)	0.88 (0.07)	0.02 (0.79)
12 Months	0.83 (0.06)	0.87 (0.07)	0.04 (0.66)
Differential change	−0.03 (0.58)	−0.02 (0.86)	0.02 (− 0.15, 0.19)
Total protein (*p* = 0.17) ^a^			
Baseline	5.16 (0.15)	5.52 (0.20)	0.36 (0.15)
12 Months	5.19 (0.15)	5.21 (0.20)	0.02 (0.94)
Differential change	0.03 (0.85)	−0.31 (0.11)	−0.34 (− 0.82, 0.14)
Dairy (p = 0.05) ^a^ *			
Baseline	2.76 (0.08)	2.88 (0.10)	0.13 (0.31)
12 Months	2.77 (0.08)	3.16 (0.10)	0.39 (< 0.01) *
Differential change	0.02 (0.69)	0.16 (< 0.01) *	0.26 (0.01, 0.51)
Total Score (*p* = 0.997) ^a, b^			
Baseline	31.44 (0.59)	32.43 (0.79)	1.00 (0.31)
12 Months	31.68 (0.61)	32.67 (0.79)	0.99 (0.32)
Differential change	0.24 (0.62)	0.24 (0.70)	0.00 (−1.54, 1.53)

^a^ *p*-value for the F-test of Time by Treatment interaction

^b^ Alcohol and fat categories are removed because the ITT and PPS analyses are the same in a GLMM setting for two time points

*Significant *p*-value (*p* ≤ 0.05)

The identification of the dairy sub-category as significantly improved related to the most attended intervention sessions (Table 5). It was a key area covered in both sessions (1 and 2) as it is one of the easiest changes to make to reduce saturated fat and total energy in Australian diets. Session 2 covered alcohol as a major contributor to empty calories and discussion focused on lower energy alternatives for situations where alcohol is consumed.

**Table 5:** Intervention attendance at each of the group sessions and the main topic of focus for each session

Session	1	2	3	4	5
Main topic	Saturated fat	Total energy	Fibre	Family eating	Stress management
Attendance	45%	40%	26%	27%	29%

## Discussion

This secondary analysis of a postnatal lifestyle intervention in women with previous gestational diabetes suggests engaged participants achieved improved dietary quality for a sub-category of the ARFS – dairy. This sub-category is directly related to the amount of saturated and total energy consumed, which for Australia is estimated to be 19% of the total saturated fat and 8% total energy intake [36]. This improvement in diet quality, while potentially significant for diabetes prevention [37], was only seen in the PPS analysis, which was defined a priori to exclude individuals who did not achieve a minimum intervention exposure or became ineligible due to pregnancy or being diagnosed with T2DM. This suggests that participant fidelity is important in achieving intervention outcomes. This modest improvement aligns with the primary outcome findings showing women in the intervention group maintaining their weight over the 12-month study period while the control group continued to gain weight at the Australian national average rate for this age group, which is 700 g per annum [38].

While an increasing number of interventions are being reported in the literature for postpartum women with previous GDM, the effect size has been small compared to diabetes prevention programs in the general population [10, 11]. The effect size of the current study on weight change is similar to the average achieved in this population [39]. A recent systematic review and meta-analysis of fifteen postpartum lifestyle interventions for women with a history of GDM showed that a variety of intervention types existed and the majority (*n* = 11) covered both physical activity and healthy eating approaches [10]. The interventions were more effective when delivered within the first six months after a child’s birth and had a duration of greater than a year [10]. It is also worth noting that the included studies used a wide variety of delivery modes including distance (phone, internet, postcards; *n* = 9); group sessions (*n* = 4); individual face-to-face contact (n = 11; *n* = 2 home visits or hospital location n = 9). This significant level of heterogeneity points to similar issues found in the MAGDA study – the intervention ‘active ingredients’ are being delivered in a wide variety of ways but a best fit is still lacking and as a result, the levels of penetration, participation and engagement are generally low [40].

The quality of the dietary intake in the women involved in the MAGDA study was low and this is an important consideration for their future health. While it is clear that this population are at-risk of developing T2DM within 5–10 years of GDM diagnosis, their dietary quality indicates that they have suboptimal intakes of key food groupings and that their dietary patterns are not well aligned with the Australian Guide to Healthy Eating [32]. Our findings support previous research done within a representative sample obtained via an Australian national survey [21]. The mean total ARFS was 30.9 (8.1 SD) in the survey of 1499 women with a history of GDM [21], which was very similar to the MAGDA cohort. Some differences were seen in the ARFS vegetable (13.5 ± 4.4 MAGDA versus 11.7 ± 4.4 survey) and alcohol (0.1 ± 0.3 MAGDA versus 1.1 ± 0.8 survey) category scores. The most poorly scored categories within both studies were nuts and legumes, grains and fruit. Changing to a dietary pattern that included a greater amount of fibre-containing breads and cereals alongside more legumes and fruit variety would see a noticeable improvement in the dietary quality of these diets. The historical and current low dietary quality scores in this population [41] have the potential to further increase their risk of chronic ill-health such as T2DM [19] and cardiovascular disease [42], which highlights the need to focus and target specific dietary changes in a meaningful way to reduce their risk.

The women within the MAGDA study reported a variety of barriers to engaging with the group education sessions [43]. The barriers of work commitments, time, cost and difficulty sourcing childcare have also been reported in other studies involving women with previous GDM [17, 44] and for women without GDM [45]. Similarly, personal barriers to changing dietary intakes exist for both women with and without previous GDM. Other aspects of health behaviour change can also be impacted in both populations such as environmental barriers to increasing physical activity [45, 46] or lacking partner support [47, 48]. Altogether the similarity that holds across women with and without GDM may mean that the barriers to engaging with lifestyle modification and programs that address them may be related to the life stage rather than the GDM itself.

Our study has several limitations; the most notable being the small effect size seen with the intervention overall, which may be related to the low level of session attendance in MAGDA [25]. The literature acknowledges that disengagement from an intervention results in less skills and coping strategies for weight reduction [49, 50] and other diabetes prevention programs have associated decreased participation with decreased weight reduction [51]. The definition of the PPS is another limitation for consideration as it did include reasons for exclusion that were not related to attendance and the criteria relating to a minimum exposure to the intervention was low (one in five of the group sessions). The findings also need to be interpreted in light of their being a secondary analysis of a RCT and that the presented analyses was not part of the original study proposal. We also acknowledge the exploratory nature of the comparisons and potential impact of inflated false discovery rate due to multiple hypothesis testing without adjustment for multiplicity. Another limitation worth considering is the relatively low metabolic risk of the MAGDA participants which was communicated to them during the first individual session as a component of their personalized risk score. It is possible that this information was interpreted as a decreased need to change and may have led to women seeing the group sessions as less important thereby decreasing their engagement with the active intervention.

## Conclusions

Our results show that a group-delivered diabetes prevention program altered some aspects of dietary quality compared to usual care in a cohort of postpartum women with previous GDM who engaged with the diabetes prevention program. The overall effect was limited by the low level of engagement with the intervention and secondary nature of this analysis. Future diabetes prevention programs should consider alternative delivery modes to increase program engagement. More research on how to increase engagement is needed and this might include designing the program with participants or delaying participation until the women have pre-diabetes and may view the program as more important to engage in.

## Data Availability

The datasets generated during and/or analyzed during the study are not publicly available due to the privacy of the participants.

## Abbreviations

ADIPS: Australasian Diabetes in Pregnancy Society
ARFS: Australian Recommended Food Score
CI: Confidence interval
FFQ: Food Frequency Questionnaire
GDM: Gestational diabetes mellitus
IADPSG: International Association of the Diabetes and Pregnancy Study Groups
ITT: Intention To Treat
MAGDA: Mothers After Gestational Diabetes in Australia
NGDR: Australian National Gestational Diabetes Register
OR: Odds ratio
PPS: Per-protocol-set
REML: Residual maximum likelihood
T2DM: type 2 diabetes
